# Winter bloom of a rare betaproteobacterium in the Arctic Ocean

**DOI:** 10.3389/fmicb.2014.00425

**Published:** 2014-08-20

**Authors:** Laura Alonso-Sáez, Michael Zeder, Tommy Harding, Jakob Pernthaler, Connie Lovejoy, Stefan Bertilsson, Carlos Pedrós-Alió

**Affiliations:** ^1^Limnology and Science for Life Laboratory, Department of Ecology and Genetics, Uppsala UniversityUppsala, Sweden; ^2^Limnological Station, Institute of Plant Biology, University of ZurichKilchberg, Switzerland; ^3^Département de Biologie, Université LavalQuébec, QC, Canada; ^4^Departament de Biologia Marina i Oceanografia, Institut de Ciències del Mar, CSICBarcelona, Spain

**Keywords:** Arctic, betaproteobacteria, biofilm, bloom, *Janthinobacterium*, rare biosphere

## Abstract

Extremely low abundance microorganisms (members of the “rare biosphere”) are believed to include dormant taxa, which can sporadically become abundant following environmental triggers. Yet, microbial transitions from rare to abundant have seldom been captured *in situ*, and it is uncertain how widespread these transitions are. A bloom of a single ribotype (≥99% similarity in the 16S ribosomal RNA gene) of a widespread betaproteobacterium (*Janthinobacterium* sp.) occurred over 2 weeks in Arctic marine waters. The *Janthinobacterium* population was not detected microscopically *in situ* in January and early February, but suddenly appeared in the water column thereafter, eventually accounting for up to 20% of bacterial cells in mid February. During the bloom, this bacterium was detected at open water sites up to 50 km apart, being abundant down to more than 300 m. This event is one of the largest monospecific bacterial blooms reported in polar oceans. It is also remarkable because *Betaproteobacteria* are typically found only in low abundance in marine environments. In particular, *Janthinobacterium* were known from non-marine habitats and had previously been detected only in the rare biosphere of seawater samples, including the polar oceans. The Arctic *Janthinobacterium* formed mucilagenous monolayer aggregates after short (ca. 8 h) incubations, suggesting that biofilm formation may play a role in maintaining rare bacteria in pelagic marine environments. The spontaneous mass occurrence of this opportunistic rare taxon in polar waters during the energy-limited season extends current knowledge of how and when microbial transitions between rare and abundant occur in the ocean.

## Introduction

Widespread reports of the large number of rare microorganisms in the environment challenge our understanding of microbial ecology and biogeography. The search for the mechanisms that maintain this “rare biosphere” has become a focus of intense research (Pedrós-Alió, 2012). Rare microbes have been proposed to have important ecological roles such as stabilizing ecosystem processes after disturbance or maintaining critical biogeochemical functions. However, a large fraction of the rare biosphere detected in high throughput sequencing studies may be composed of dead microbes, or dormant/inactive microbial species. These non-dividing microbes are prone to extinction over time, but they may act as a reservoir of taxa able to proliferate under specific conditions, as suggested by the “seed bank” hypothesis, with key ecological implications (Lennon and Jones, 2011). While dormancy is increasingly assumed to play a key role in fostering and maintaining microbial diversity, testing hypotheses related to rare and dormant microbes in natural ecosystems is still challenging.

By comparing ratios of 16S ribosomal RNA to 16S ribosomal RNA genes (from RNA and DNA, respectively) of rare and abundant bacterial taxa, some rare microbes appear to be highly active in aquatic environments (Jones and Lennon, 2010; Campbell et al., 2011). A likely explanation for this observation is that microbial rank abundances are dynamic and characterized by transitions between dormant and active states (Lennon and Jones, 2011). Examples of microbial shifts from rare to abundant are typically found in experimental manipulations such as bottle incubations (Fuchs et al., 2000), after the addition of specific organic compounds (Teira et al., 2007) or under changes in experimental conditions (Bouvier and del Giorgio, 2007; Sjöstedt et al., 2012). Yet, there are extremely few examples of such microbial transitions taking place in the environment (Piccini et al., 2006).

In the ocean, transient blooms of rare Flavobacteria populations have been found associated with phytoplankton blooms in highly productive waters (Teeling et al., 2012). Episodic increases in the abundance of fast-growing *Gammaproteobacteria* such as *Glaciecola* (Alonso-Sáez et al., 2007) or *Alteromonas* (Bano and Hollibaugh, 2002) have also been reported, with no clear link with any obvious environmental trigger. However, reports of temporal shifts of bacterial taxa from rare to abundant are still scarce in the surface ocean. A pyrosequencing study in Arctic waters did not find evidence for such transitions (Kirchman et al., 2010), and a 6-year time-series study in the English Channel only reported a single massive bloom of a rare species (*Vibrio* sp.) at a time point coincident with maximum concentrations of nitrogen, carbon and chlorophyll (Gilbert et al., 2012).

Such transitions are by definition ephemeral. Therefore, the monthly resolution of most temporal studies may largely miss bacterial bloom events if they take place over timescales spanning days to weeks. Thus, more highly resolved temporal sampling programmes could be critical for detecting blooms of rare taxa. Finding out what types of microorganisms are prone to increase rapidly, how often these blooms occur *in situ*, and identifying environmental thresholds for these transitions would greatly enhance our understanding of the ecology of rare microorganisms. In the present study, we illustrate this case by reporting a transient bloom of a rare betaproteobacterium in Arctic seawater, detected by weekly sampling during winter under the ice cover.

## Materials and methods

**Figure 1:**
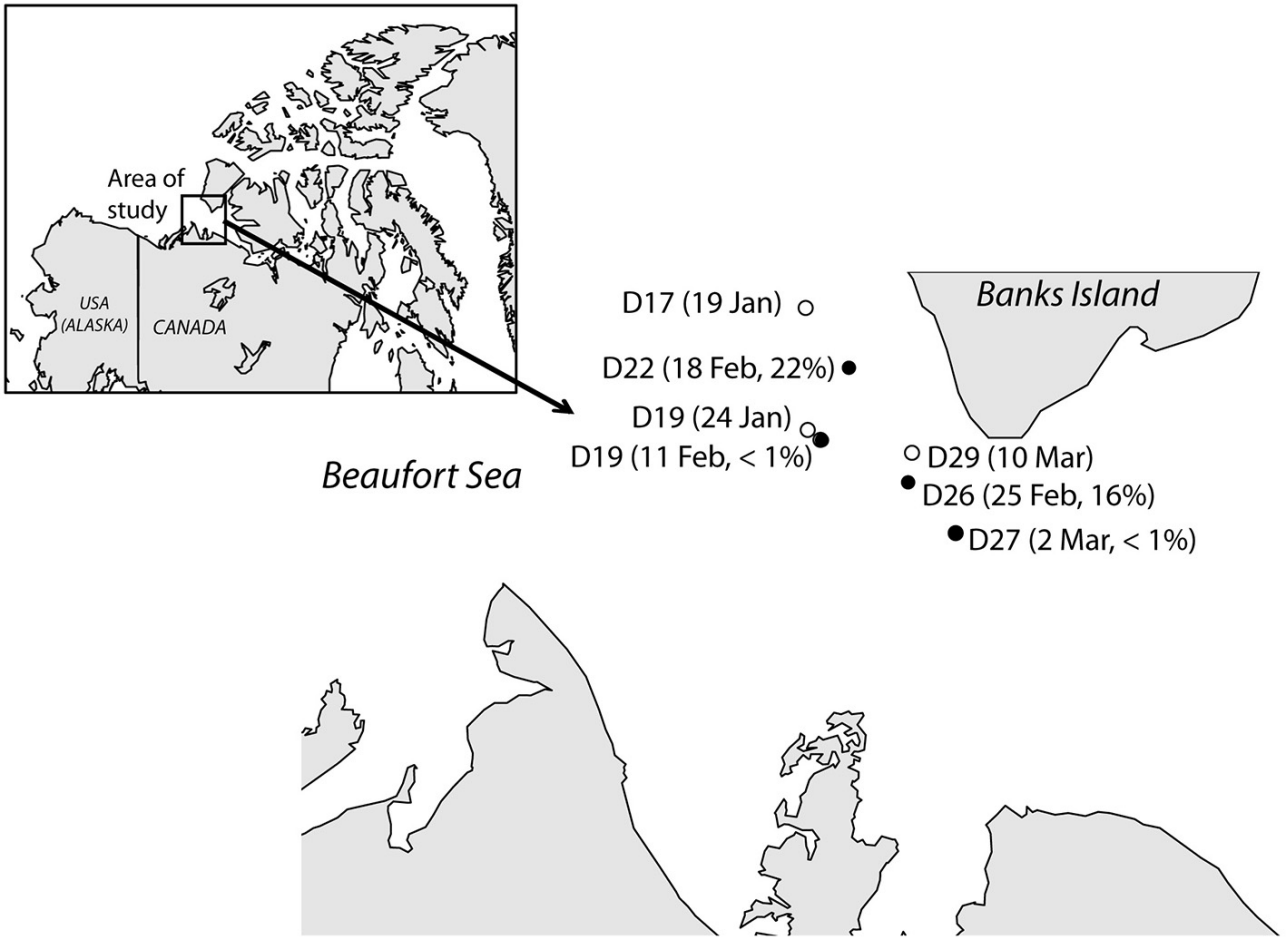
**Map showing the location of the stations in this study and the dates when they were sampled in 2008**. Two different samples were collected at station D19 while the research vessel was passively drifting with the ice. Filled dots represent stations where *Janthinobacterium* cells were detected *in situ* by CARD-FISH, and their contribution (in percentage of total cells) are shown.

Seawater samples down the depth profile were collected weekly in different stations from the Amundsen Gulf, Western Arctic, from January to March 2008 on board the CCGS Amundsen (Figure 1). Samples for Catalyzed Reporter Deposition Fluorescence *in situ* Hybridization (CARD-FISH) and DNA analyses were collected directly from 12-L Niskin-type bottles mounted on a Carousel Rosette. Profiles of temperature and salinity were obtained using a SeaBird 911 + CTD mounted on the rosette, with additional probes for oxygen and chlorophyll fluorescence. Concentrations of nitrate were determined using standard colorimetric methods (Grasshoff et al., 1999) adapted for the AutoAnalyzer 3. Surface samples analyzed in this study were collected at a depth of 12 m to avoid any potential ship-associated contamination. We also analyzed an additional seawater sample collected at 5 m depth on February 5th through a hole in the ice ca. 450 m upstream from the ship.

Microbial biomass for DNA analysis was collected by sequentially filtering 7 L of sample through a 3-μm pore size polycarbonate filter (Poretics) and a 0.2-μm pore size Sterivex filter unit using a peristaltic pump. Contents on both filters were preserved with 1.8 mL of lysis buffer (50 mM Tris-HCl pH 8.3, 40 mM EDTA pH 8.0, 0.75 M sucrose) and kept at −80°C. A seawater sample collected in February 18th at station D22 (71°18.649 N, −124°29.798 W) was selected for DNA cloning and sequencing. Two clone libraries were generated with samples collected in the size-fraction above and below 3 μm (large and small size-fraction, respectively). Nucleic acids were extracted using a standard salt protocol and 16 S rRNA genes were amplified by PCR with the universal primers 8F and 1492R. DNA extraction, cloning and sequencing were performed as described in Harding et al. (2011). Sixty-nine clones were analyzed in each clone library. Sequences were checked using the Chimera check software at Ribosomal Data Project (RDP) and chimeras were removed from further analysis. The remaining sequences were manually trimmed and taxonomically classified using the RDP Classifier (Wang et al., 2007). Sequences affiliated with *Janthinobacterium* were aligned using MUSCLE, and the alignment was manually edited using Jalview v. 2.8.0b1. Maximum-likelihood (ML) analysis was carried out with RAxML v. 7.5.6 (Stamatakis et al., 2005). The ML tree was identified following 300 alternative runs on distinct starting trees under the GTR + gamma + I model. A bootstrap analysis was also conducted in RAxML using 1000 replicates. Nucleotide sequences have been deposited in GenBank (Accession numbers KJ365318–KJ365399).

Samples for CARD-FISH (Pernthaler et al., 2004) were fixed with formaldehyde (3.7% final conc., overnight at 4°C), then filtered onto 0.22-μm pore size polycarbonate filters and frozen (−20°C) until analysis. Cells were permeabilized with lysozyme (1 h) and achromopeptidase (30 min) at 37°C. A specific oligonucleotide probe was designed (Jan64 5′-CAAGCTCCGTGCTGCCGTT-3′) that targets a large number (>2000) of sequences affiliated with cultured and uncultured *Janthinobacterium* sp., including the genotypes from the Arctic clone libraries. An online ProbeCheck analysis (Loy et al., 2008) against the current SILVA reference database (Pruesse et al., 2007) revealed that only 16 non-target sequences matched this probe even if 3 base pair mismatches were accounted for. The probe Jan64 was applied for sample analysis using 55% formamide in the hybridization buffer. Additionally, specific probes for Alphaproteobacteria (Alf968), Gammaproteobacteria (Gam42a), Bacteroidetes (CF319a), and the general probe set for Bacteria (Eub338-II-III) were used as previously described (Alonso-Sáez et al., 2007). The filter sections were counter-stained with DAPI (1 μg mL^−1^). At least 300 DAPI cells were counted manually in a minimum of 10 fields.

For MAR-CARD-FISH (Microautoradiography combined with CARD-FISH) analysis, surface samples (20 mL) were incubated with [^3^H]-leucine (0.5 nM, Perkin Elmer, NET460A005, 140 Ci/mmol) in the dark, in ice-cold seawater for ca. 8 h. After incubation, samples were fixed with formaldehyde (1.8%, overnight at 4°C) and filtered onto 0.2-μm polycarbonate filters (Millipore). Filters were hybridized following the CARD-FISH protocol, and subsequently processed for microautoradiography as previously described (Alonso-Sáez et al., 2008). Microautoradiography preparations were imaged by fully automated microscopy (Zeder and Pernthaler, 2009; Zeder et al., 2011) using the automated image analysis software ACMEtool Tool (www.technobiology.ch). Since aggregates were present as monolayers and the nucleic acid stain signals of individual cells were well separated, the quantification of their abundance in the samples (i.e., cells hybridized with probe Jan64 from total DAPI-stained cells) was possible by this automated procedure (Supplementary Figure 1).

## Results

**Figure 2:**
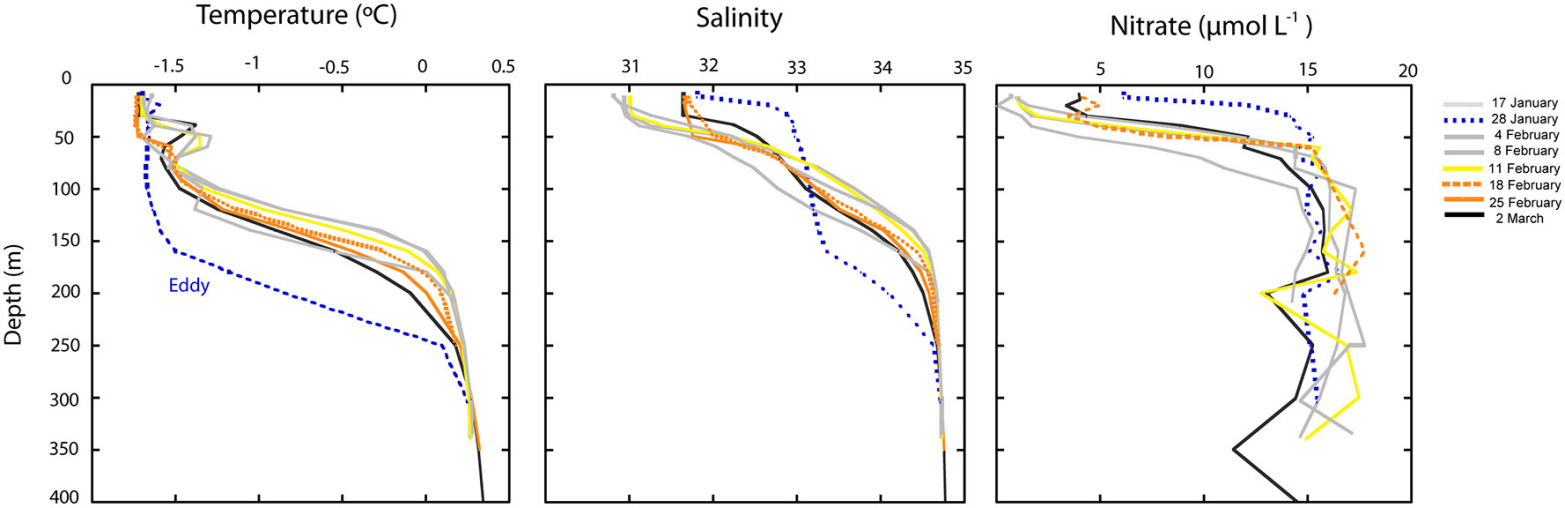
**Depth profiles of temperature, salinity and concentration of nitrate during the study**. The dotted blue line corresponds to the temperature profile on January 28th, when an oceanographic eddy crossed the Arctic area of study. The yellow line correspond to the station where *Janthinobacterium* was first detected *in situ* and orange lines correspond to the stations where maximal abundances of *Janthinobacterium* were found by CARD-FISH.

**Figure 3:**
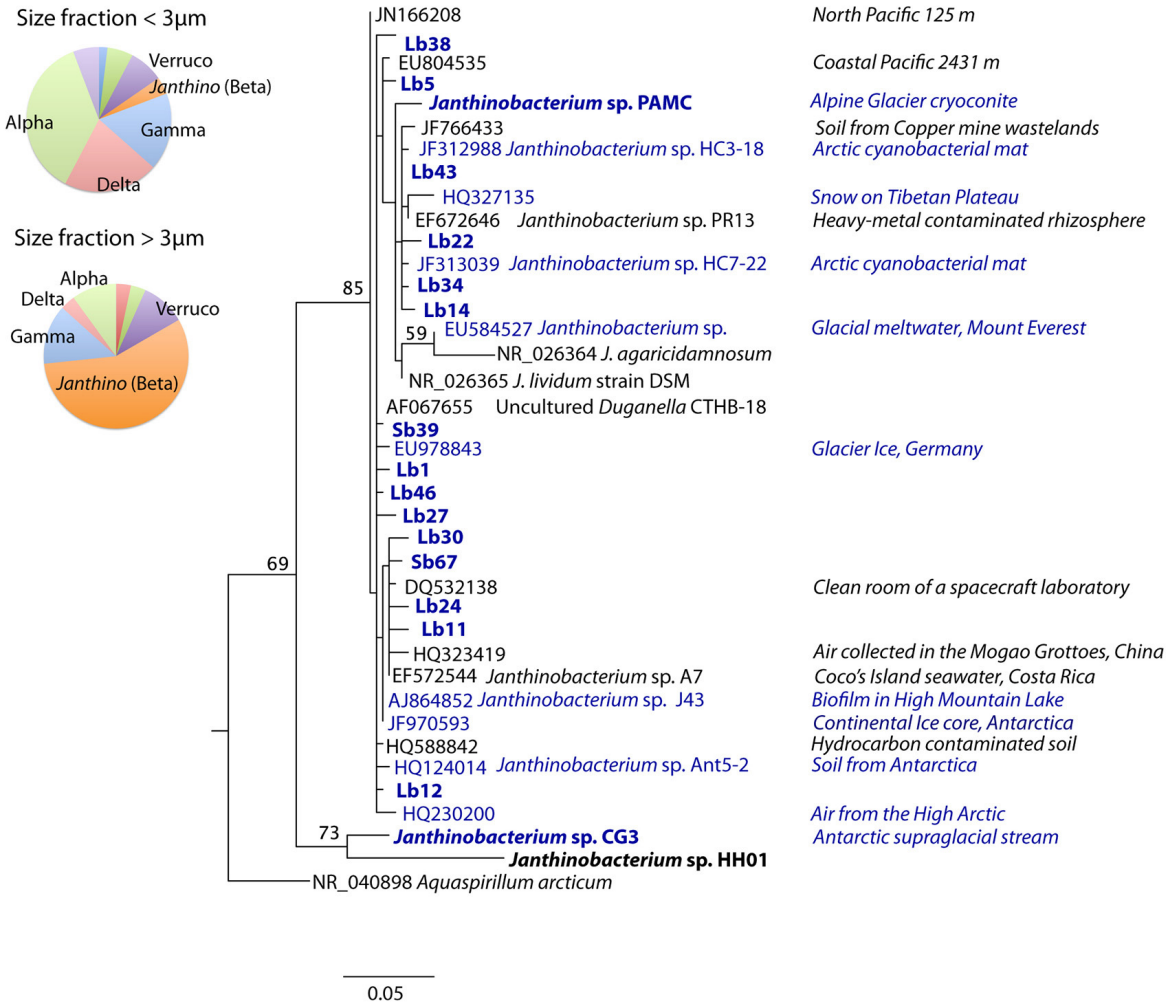
**Maximum-likelihood phylogenetic tree of sequences affiliated with *Janthinobacterium* including the environmental clones retrieved from Arctic seawater (highlighted in bold) from the clone libraries constructed with DNA from different size-fractions**. *Janthinobacterium* representatives with their genomes sequenced have also been highlighted in bold, and those isolated from cold environments (including ice, snow, glacier melt waters and high-mountain lakes) appear in blue. Isolation sources are shown to the right. The tree was rooted with a clade containing sequences of *Burkholderia* spp. (full tree is shown in Supplementary Figure 4), and bootstrap values based on 1000 replicates (>50%) are indicated on the branches. The pie charts show the proportion of clones affiliated with different bacterial phylogenetic groups as identified by the RDP classifier in the small and large size-fraction clone libraries (Verruco: Verrucomicrobia, Alpha: Alphaproteobacteria, Beta: Betaproteobacteria, Gamma: Gammaproteobacteria, Delta: Deltaproteobacteria, *Janthino*: *Janthinobacterium*).

Samples for CARD-FISH and DNA analyses were collected weekly under the ice in Arctic coastal waters from January to March 2008 (Figure 1). During this period the microorganisms experienced extremely low light availability and the surface water temperature (−1.7°C) was close to freezing point (Figure 2). Clone libraries from a sample collected on February 18th recovered an unusually high abundance of sequences affiliated with a single betaproteobacterial species (*Janthinobacterium* sp.). These sequences were mostly recovered in the size-fraction >3 μm accounting for 57% of the clones, and they formed a tight cluster (>99% similarity) closely related with the isolate *Janthinobacterium lividum* and the genome-sequenced psychrotolerant *Janthinobacterium* sp. PAMC 25,724 (Figure 3). Based on the sequencing information, a specific oligonucleotide probe targeting a large diversity of *Janthinobacterium*, including the sequences from the Arctic clone libraries, was designed and used to follow the winter temporal dynamics of this population in surface Arctic waters.

**Figure 4:**
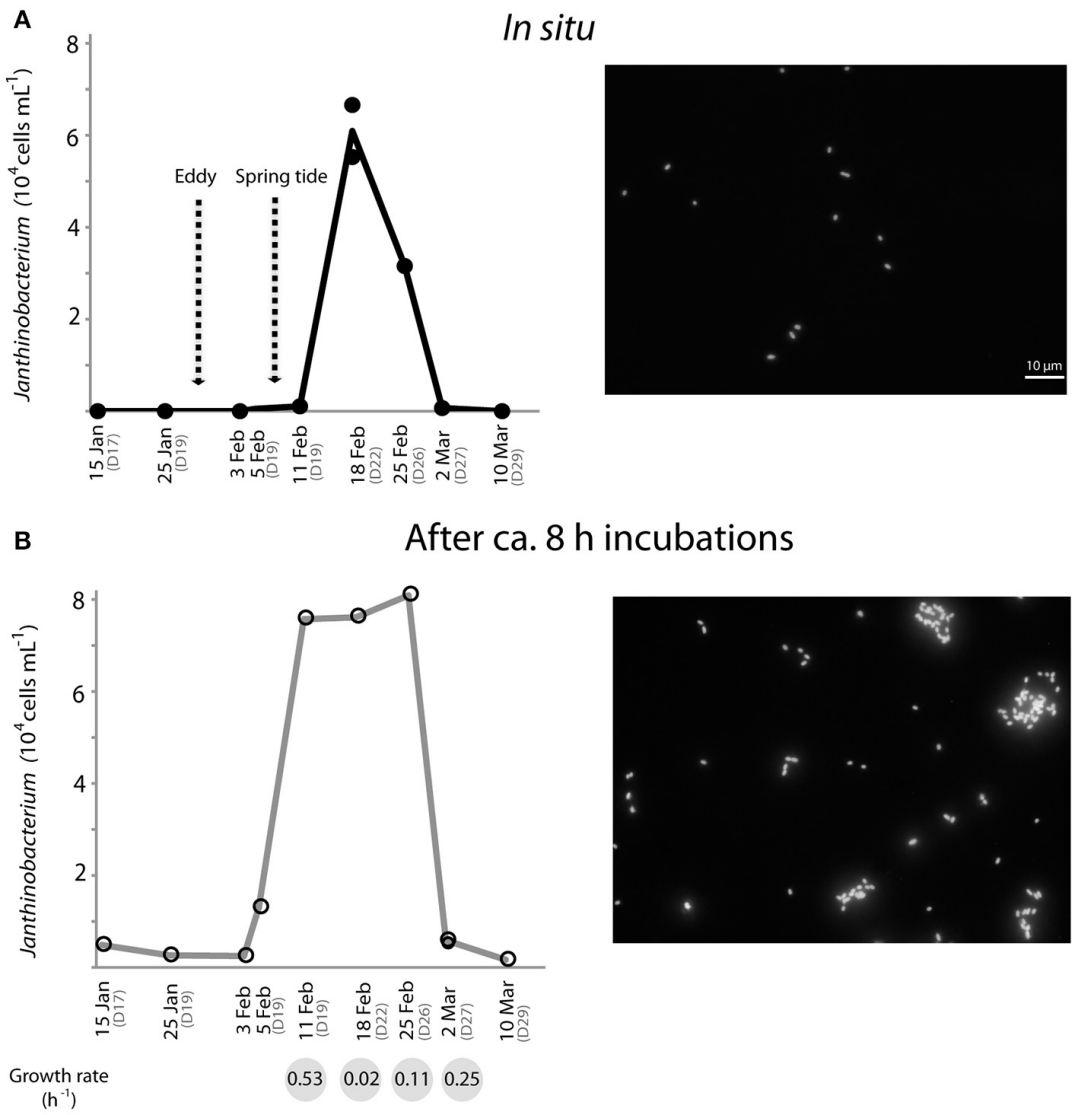
**Temporal dynamics of the *in situ* abundance of *Janthinobacterium* (probe Jan64) in surface Arctic seawater as analyzed by CARD-FISH (A), and abundances reached by the same population after 8 h incubations (for MAR-CARD-FISH analysis) of the corresponding environmental samples (B)**. Fluorescence microscope images of Arctic *Janthinobacterium* cells *in situ* (where they appeared mostly as single-cells) and in the MAR-CARD-FISH incubations (where they formed mucilaginous aggregates) are shown on the right side. The arrows in **(A)** indicate the day that an eddy crossed the Arctic area of study and the presence of strong spring tides due to the full moon on February 7th. Specific growth rates (h^−1^) of *Janthinobacterium* estimated in the incubations from February 11th to March 2nd are shown in the gray circles below the graph in **(B)**. All samples were collected onboard at 12 m depth, except for the MAR-CARD-FISH sample from February 5th, which was collected at 5 m depth through a hole in the ice. Replicate counts are shown when available.

**Figure 5:**
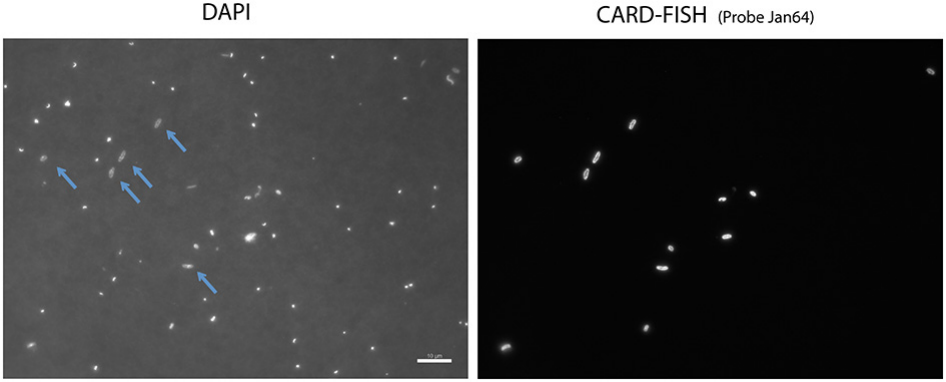
**Microscope images of the Arctic *Janthinobacterium* population (right) and all cells stained with DAPI (left) in a sample collected on February 25th**. Some of the hybridized cells are highlighted with arrows in the DAPI images.

**Figure 6:**
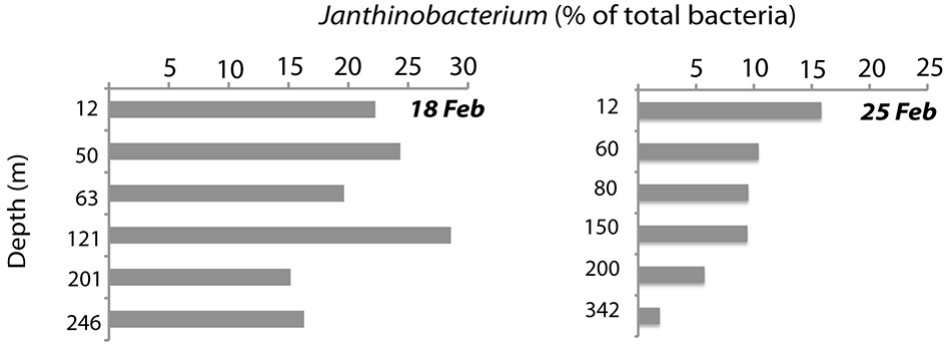
**Abundance of *Janthinobacterium* down the depth profile on the two dates when maximal abundances were found (February 18th and 25th), as analyzed by CARD-FISH**.

*Janthinobacterium* were below CARD-FISH detection levels in samples collected in January and early February (Figure 4A). These microorganisms were first detected on February 11th, when they contributed <1% of bacterial cells, and subsequently increased over the two following weeks with maximum concentrations on February 18th (22% of surface bacteria). The targeted cells appeared mostly as single individuals or within small aggregates (Figure 4A) and compared to other ambient marine bacteria they were unusually large (Figure 5). The length of *Janthinobacterium* cells was 1.48 ± 0.76 μm (*n* = 268) and 2.13 ± 1.12 μm (*n* = 89) in surface samples collected on February 18th and 25th, respectively (Figure 5). For comparison, the average length of other ambient bacteria in the samples was 0.86 ± 0.58 μm. High abundances of this population were detected in samples collected ca. 50 km apart (stations D22 and D26, Figure 1), indicating a non-local spatial distribution of this bloom. Moreover, high abundances of *Janthinobacterium* were also found in deeper samples at the time of the bloom (Figure 6), reaching 28% of cells below 100 m on February 18th. In early March (March 2nd and March 10th), when the ship returned to a location close to where the bloom had been first discovered (station D29, Figure 1), *Janthinobacterium* were again rare (i.e., below 1% of cells).

The single-cell *in situ* activity of *Janthinobacterium* in the uptake of an amino acid (leucine) from January to March was analyzed by MAR-CARD-FISH. We found that the Arctic *Janthinobacterium* were not actively incorporating leucine (<1% active cells) at any of the sampling points. Yet, increases in the total cell abundance of *Janthinobacterium* were found during the relatively short MAR-CARD-FISH incubations, by comparing their abundance *in situ* (in samples immediately fixed after collection) and after ca. 8 h of incubation. These results confirm their capacity to grow in Arctic winter seawater. *Janthinobacterium* were detected at low abundance (<1% of DAPI cells) in the incubations performed in January and late March (March 26th, Figure 4B). However, a steady increase in their abundance was observed in the incubations from the 5th to the 25th of February, while the contribution of other major bacterial groups did not show drastic changes (Supplementary Figure 2). Estimated specific growth rates of *Janthinobacterium* in the incubations were higher at times of low *in situ* abundance as compared to the time of the bloom, suggesting that the population had reached its carrying capacity (Figure 4). Interestingly, while growing in the vials, the Arctic *Janthinobacterium* population developed mucilaginous biofilm-like aggregates, indicating their ability to switch between free-living and attached lifestyles (Figure 4B and Supplementary Figure 3).

Over the sampling period, temperature and salinity profiles varied in the region, particularly in surface waters (Figure 2). The most conspicuous change in physico-chemical parameters occurred at the end of January and was due to water column mixing associated with the passage of a cold-core eddy (Figure 2). Nutrient concentrations significantly increased in surface waters during this event (Figure 2), and after ca. a week (4th of February), the water column was again stratified. During this post-eddy period *Janthinobacterium* were first detected in MAR-CARD-FISH incubations and *in situ* (5–11th February). The following weeks temperature and salinity profiles did not show substantial changes, suggesting that the water masses were relatively stable. In the upper 50 m, salinity and nitrate concentration were elevated at the stations where maximum abundances of *Janthinobacterium* were found (D22 and D26, sampled on the 18th and 25th February, respectively), and also at station D27 (sampled on 2nd March), when *Janthinobacterium* were again rare.

## Discussion

*Janthinobacterium* have been found in numerous habitats and have attracted substantial interest for biotechnological applications (Du et al., 2007; Shi et al., 2008; Johnsen et al., 2010). Interestingly, isolates of *J. lividum* and the strain PAMC 25,724, closely related to the Arctic population studied here, are able to produce violacein (Pantanella et al., 2007; Kim et al., 2012), a pigment with anti-grazing properties that may provide a survival advantage in the environment (Matz et al., 2004). We present the first report of a natural bloom of *Janthinobacterium* and, to our knowledge, also the first report of a betaproteobacterium bloom in seawater. While abundant and active in freshwater systems (Glöckner et al., 1999; Salcher et al., 2013), the concentration of Betaproteobacteria sharply decreases when exposed to marine salinities (Cottrell and Kirchman, 2003; Garneau et al., 2006). A few marine betaproteobacterial taxa have shown high activity, such as the OM43 clade, but their abundance is usually low, rarely exceeding 2% of cells (Morris et al., 2006; Sowell et al., 2011). The large proportion (up to 22% of total cells) and spatial extent of the *Janthinobacterium* bloom found in this study indicates the capacity of this rare microorganism to proliferate under particular conditions. Ribosomal gene sequences with similarity ≥94% to *J. lividum* were found in 15 stations of the Global Ocean Sampling (GOS, search using the ncRNA database, http://portal.camera.calit2.net), but only seven hits with similarity >97% were found. Some sequences affiliated with *Janthinobacterium* were also found in Arctic and Antarctic waters (VAMPS database, http://vamps.mbl.edu/), but being always rare (<0.01%).

Despite their low abundances, the wide range of environments where *Janthinobacterium* has been found is striking (Figure 3). In an early study, a phylotype closely related to *J. lividum* (99% similarity for the 16S rRNA gene) was found in a negative control of a DNA extraction (AF067655, “uncultured *Duganella* clone CTHB-18”) leading to the suggestion that it might be a PCR contaminant (Tanner et al., 1998). This possibility can be ruled out in our study since *Janthinobacterium* were detected at high concentrations both *in situ* and in seawater incubations by the PCR-independent CARD-FISH approach. At the global scale, *Janthinobacterium* have been retrieved in soils, lakes, seawater, and the cold biosphere (Gillis and De Ley, 2006), including mountain glaciers, polar ice, and snow, where high abundances have been occasionally found (Segawa et al., 2005). Their cosmopolitan distribution suggests a very effective dispersal capacity and implies physiological flexibility to colonize new environments. Interestingly, *Janthinobacterium* have also been found in dust and air, including some samples from the High Arctic (HQ230200, Harding et al., 2011), indicating that aerial transport may be a major dissemination pathway for this cosmopolitan microbe.

The fact that *Janthinobacterium* were abundant down the depth profile during the bloom despite stratification, raises the question about the mechanisms that would have facilitated their distribution throughout the water column. Actually, oceanographic eddies actively traveled under the ice in this region during the winter of 2008. On January 28th, a cold-core eddy crossed the area of study, followed by strong tidal currents due to a new moon on Feburary 7th. Both phenomena generated turbulence and enhanced vertical mixing (Gratton et al., 2012). Remarkably, an increase in the reproductive activity of small copepods was observed at the same time, consistent with an unusual winter increase in the biological production of some components of the Arctic marine food web (Darnis et al., 2012). The latter authors suggested that the passage of eddies may have brought particulate organic matter susceptible to exploitation by attached bacterial communities, which may have subsequently been exploited for food by small detritivorous copepods.

Evidence of the use of pathways for exploiting high-molecular weight compounds such as humic acids or carbon polymers has been found in the *J. lividum* isolate OW6/RT-3 (Freese et al., 2010), and in the genome of the psychrotrophic *Janthinobacterium sp*. HH01 (Hornung et al., 2013), respectively. While we did not test the activity of Arctic *Janthinobacterium* on polymers, they did not actively take up the amino acid leucine, which is a typical small monomer. In addition, seawater incubations carried out during the *Janthinobacterium* bloom showed that this microbe formed aggregates (Figure 4B), which favors the use of exoenzymes needed to break down more complex organic matter (Huston and Deming, 2002). Overall these lines of evidence are consistent with the possibility of *Janthinobacterium* profiting from an influx of complex substrates (Freese et al., 2010).

The growth of *Janthinobacterium* in the relatively short (8 h) incubations in ice-cold waters provided additional clues about their activity and lifestyle. First, large increases in total cell numbers occurred in the incubations carried out between early February and March, with an unusually short doubling time of 1.3 h on February 11th. Comparably fast growth rates have been reported for some *Janthinobacterium* isolates with a doubling time of 1.1 h (Hornung et al., 2013). In a cold non-marine environment (mountain snow) rapid temporal increases of *Janthinobacterium* abundance (ca. 10^5^-fold by quantitative PCR) have been reported during the melting season, preceding the growth of snow algae (Segawa et al., 2005). These results support the view that this psychrophilic group, able to produce bursts of growth, does not rely on labile photosynthetic products as substrates. The different cell abundance reached by *Janthinobacterium* in the incubations from January to March seemed consistent with a temporal change in their activity. The lower activity outside of the blooming period (Figure 4) may be due to a lack of availability of suitable substrates. An alternative explanation may have been a release from top-down pressures. In a previous study with samples off the coast of Oregon, rare members of betaproteobacteria appeared to be tightly controlled by viruses (Bouvier and del Giorgio, 2007). Changes in physico-chemical conditions associated with the passage of eddies may have disrupted interactions between *Janthinobacterium* and their grazers, allowing this group to bloom. Unfortunately, species-specific viral data is not available, and there is currently no evidence supporting such viral control.

The production of biofilms associated with autoinducing signals (i.e., quorum sensing) has been described for the isolate *J. lividum*, and appears to be regulated by changes in the carbon source (Pantanella et al., 2007). The latter authors postulated that alternations between free-living and attached life-styles could represent a response to environmental stress, and a key factor in the survival of *J. lividum* and their ability to colonize new environments. The Arctic *Janthinobacterium* population developed mucilaginous biofilm-like aggregates possibly triggered by confinement in vials during MAR-CARD-FISH incubations (Figure 4B). While the presence of mechanisms for quorum sensing in psychrophiles has been largely unexplored (Montgomery et al., 2013), our results suggest that biofilm formation is a strategy for some rare Arctic marine bacteria, and support the hypothesis that signaling compounds may have a role in dormant microbes (Epstein, 2009).

Whether the *Janthinobacterium* population was part of the local “seed bank” of Arctic waters or whether they were inocula from another ecosystem such as ice, snow or air before the beginning of sampling, cannot be confirmed based on available data. However, the possibility of advection of a large population of *Janthinobacterium* cells from another region rather than an *in situ* bloom is unlikely due to several reasons. First, the bloom was not local (Figure 1) and occurred throughout the water column. Despite some variability in surface samples, the physico-chemical properties of the water masses were relatively constant shortly before, during and after the bloom (from the beginning of February to March, Figure 2). Second, the fast growth rates detected in the incubations demonstrate that *Janthinobacterium* could actively grow in Arctic winter waters outcompeting other Arctic marine bacterial groups at low temperatures. As mentioned earlier, a plausible explanation to their mass occurrence in mid February involves the passage of an oceanographic eddy facilitating their distribution with depth and carrying a supply of particulate carbon that may have promoted their growth over the following weeks.

Finally, in a recent comparison of the marine bacterial composition between poles, *Betaproteobacteria* were always rare, but their contribution was higher in the Arctic as compared to the Antarctic Ocean (Ghiglione et al., 2012). While it has been suggested that such a difference is due to the higher freshwater inputs to the Arctic Ocean, we hypothesize that the ability of some Arctic *Betaproteobacteria* to produce episodic bursts of growth could also contribute to their higher abundance in Arctic marine “seed banks.”

In summary, our study provides one of the first examples of how a widespread member of the rare biosphere (*Janthinobacterium* sp.) produces a transient bloom in oceanic waters. The fact that the environmental setting of the bloom was the Arctic during winter, a cold and energy-limited environment, broadens current knowledge of how and when transitions of bacterial taxa between rare and abundant occur in the ocean. It is also the first report of a transient bloom of a single betaproteobacterial species to become highly abundant in seawater (over 20% of total counts). *Janthinobacterium* have a widespread distribution and the capability to survive and colonize new environments, features that presumably characterize some members of the rare biosphere that appear to be persistent in oceanic seed-banks (Gibbons et al., 2013). Their ability to produce mucilaginous (biofilm-like) aggregates and to switch between free-living and attached lifestyles, may contribute to the survival of this and other rare microbes, and to their capacity to produce ephemeral blooms. Finally, our results highlight the fact that low-frequency sampling can overlook relevant microbial events such as the blooms of rare microbes, with potentially important impacts on our understanding of oceanic biogeochemistry.

### Conflict of interest statement

The authors declare that the research was conducted in the absence of any commercial or financial relationships that could be construed as a potential conflict of interest.
