# Supplementary material for: Winter bloom of a rare betaproteobacterium in the Arctic Ocean
Source: Front Microbiol. 2014 Aug 20;5:425. doi: 10.3389/fmicb.2014.00425 (PMC4138443; doi:10.3389/fmicb.2014.00425)
Supplement: Supplementary file 1 [file Presentation1.PDF]

Supplementary Material

Winter bloom of a rare betaproteobacterium in the Arctic Ocean

Laura Alonso-Sáez[*,1], Michael Zeder[2], Tommy Harding[3], Jakob Pernthaler[2], Connie Lovejoy[3], Stefan Bertilsson[1], Carlos Pedrós-Alió[4]

[1] Limnology and Science for Life Laboratory, Department of Ecology and Genetics, Uppsala University, Uppsala, Sweden
[2] Limnological station, Institute of Plant biology, University of Zurich, Kilchberg, Switzerland
[3] Département de Biologie, Université Laval, Québec, Canada
[4] Departament de Biologia Marina i Oceanografia, Institut de Ciències del Mar, CSIC, Barcelona, Spain

***Correspondence:** Laura Alonso-Sáez, AZTI-Tecnalia, Marine Research Unit, Txatxarramendi Irla, 48395 Sukarrieta, Spain.
laura@azti.es

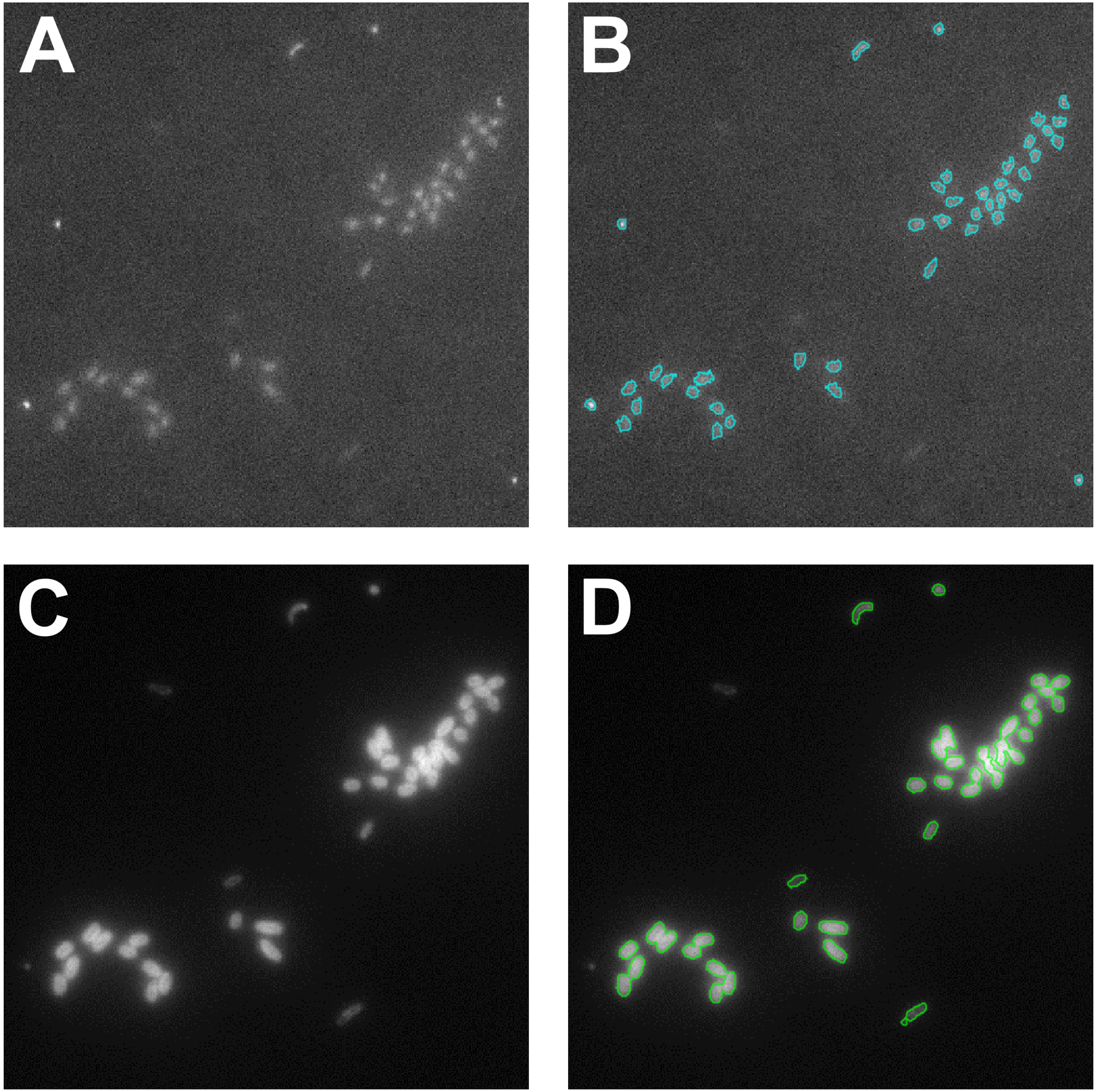

Supplementary Figure 1

Epifluorescence microscopy images of DAPI-stained (A) and CARD-FISH hybridized (C) *Janthinobacterium* cells, and their detection by the automated image analysis software ACMETool (B and D).

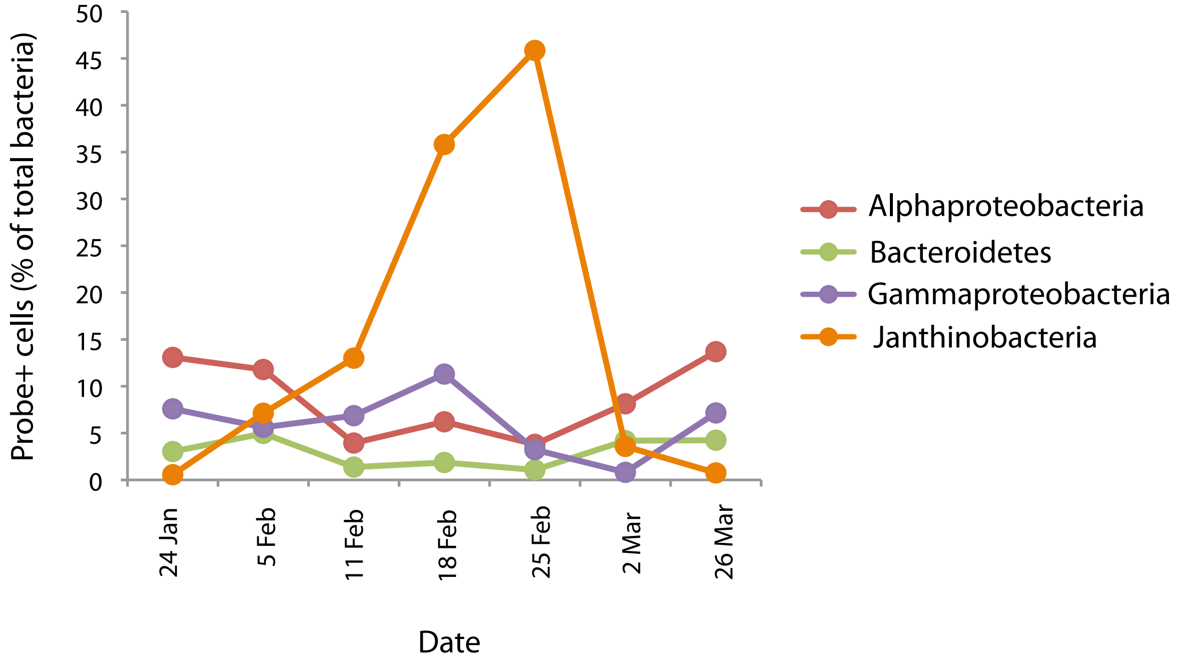

Supplementary Figure 2

Temporal changes in the contribution of different bacterial groups (Alphaproteobacteria, Bacteroidetes, Gammaproteobacteria and *Janthinobacterium*) in MAR-CARD-FISH incubations performed from 24th January to 26th March 2008.

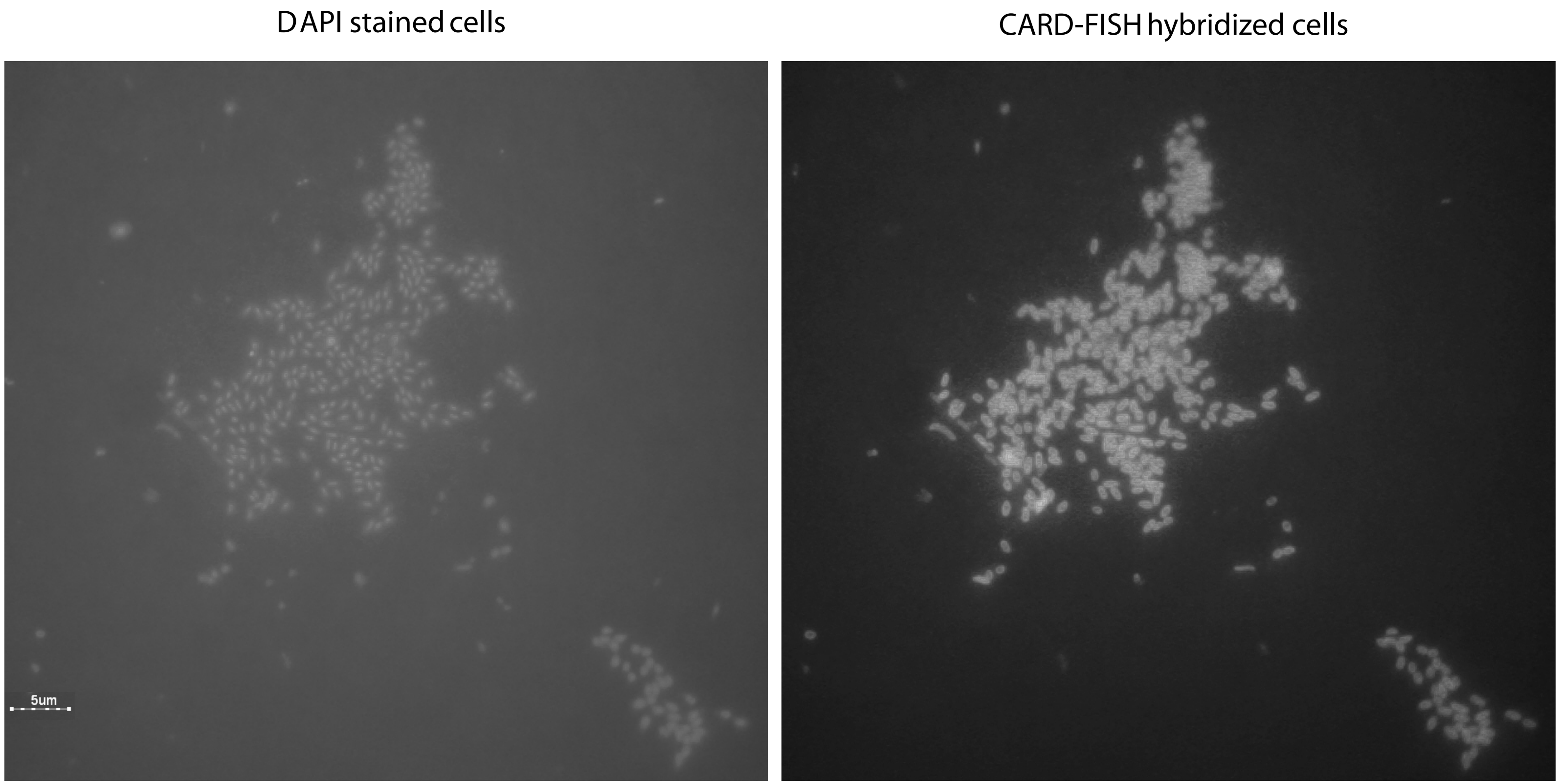

Supplementary Figure 3

Fluorescence microscopy images of the Arctic *Janthinobacterium* populations forming monolayer biofilm-like aggregrates when growing in MAR-CARD-FISH incubations.

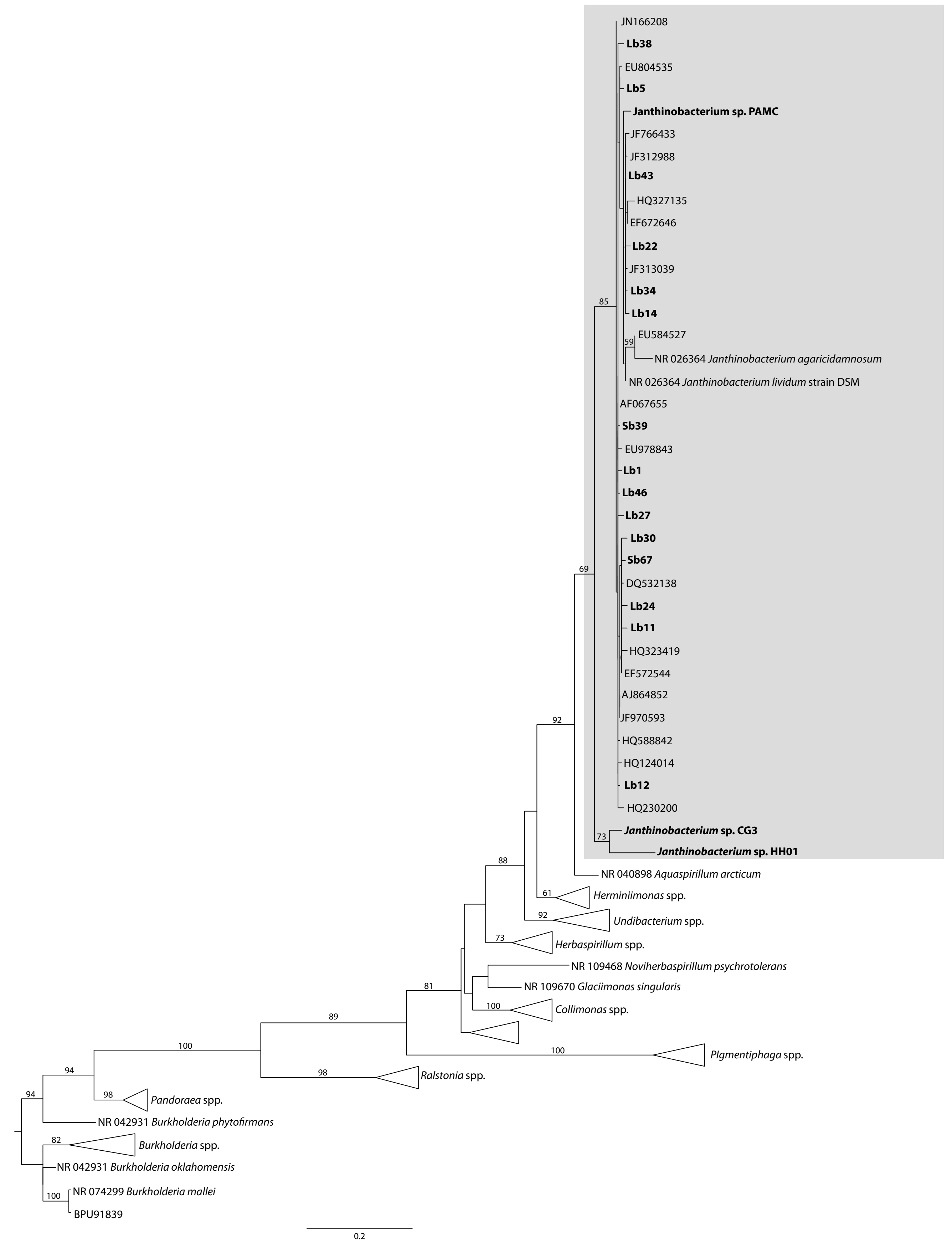

Supplementary Figure 4

Maximum-likelihood phylogenetic tree showing the relationship of 16S rRNA genes of the janthinobacteria retrieved in the Arctic. The *Janhinobacterium* clade has been highlighted. Bootstrap values based on 1000 replicates (>50%) are indicated on the branches.
